# Treatment Response and Mortality among Patients Starting Antiretroviral Therapy with and without Kaposi Sarcoma: A Cohort Study

**DOI:** 10.1371/journal.pone.0064392

**Published:** 2013-06-05

**Authors:** Mhairi Maskew, Matthew P. Fox, Gilles van Cutsem, Kathryn Chu, Patrick MacPhail, Andrew Boulle, Matthias Egger, for IeDEA Southern Africa

**Affiliations:** 1 Health Economics and Epidemiology Research Office, Department of Internal Medicine, School of Clinical Medicine, Faculty of Health Sciences, University of the Witwatersrand, Johannesburg, South Africa; 2 Center for Global Health and Development, Boston University, Boston, Massachusetts, United States of America; 3 Department of Epidemiology, Boston University School of Public Health, Boston, Massachusetts, United States of America; 4 Médecins Sans Frontières, Cape Town, South Africa; 5 Centre for Infectious Disease Epidemiology and Research (CIDER), University of Cape Town, Cape Town, South Africa; 6 South Africa Medical Unit (SAMU), Médecins Sans Frontières, Johannesburg, South Africa; 7 Institute of Social and Preventive Medicine (ISPM), University of Bern, Bern, Switzerland; Hannover Medical School, Germany

## Abstract

**Background:**

Improved survival among HIV-infected individuals on antiretroviral therapy (ART) has focused attention on AIDS-related cancers including Kaposi sarcoma (KS). However, the effect of KS on response to ART is not well-described in Southern Africa. We assessed the effect of KS on survival and immunologic and virologic treatment responses at 6- and 12-months after initiation of ART.

**Methods:**

We analyzed prospectively collected data from a cohort of HIV-infected adults initiating ART in South Africa. Differences in mortality between those with and without KS at ART initiation were estimated with Cox proportional hazard models. Log-binomial models were used to assess differences in CD4 count response and HIV virologic suppression within a year of initiating treatment.

**Results:**

Between January 2001–January 2008, 13,847 HIV-infected adults initiated ART at the study clinics. Those with KS at ART initiation (n = 247, 2%) were similar to those without KS (n = 13600,98%) with respect to age (35 vs. 35yrs), presenting CD4 count (74 vs. 85cells/mm^3^) and proportion on TB treatment (37% vs. 30%). In models adjusted for sex, baseline CD4 count, age, treatment site, tuberculosis and year of ART initiation, KS patients were over three times more likely to have died at any time after ART initiation (hazard ratio[HR]: 3.62; 95% CI: 2.71–4.84) than those without KS. The increased risk was highest within the first year on ART (HR: 4.05; 95% CI: 2.95–5.55) and attenuated thereafter (HR: 2.30; 95% CI: 1.08–4.89). Those with KS also gained, on average, 29 fewer CD4 cells (95% CI: 7–52cells/mm^3^) and were less likely to increase their CD4 count by 50 cells from baseline (RR: 1.43; 95% CI: 0.99–2.06) within the first 6-months of treatment.

**Conclusions:**

HIV-infected adults presenting with KS have increased risk of mortality even after initiation of ART with the greatest risk in the first year. Among those who survive the first year on therapy, subjects with KS demonstrated a poorer immunologic response to ART than those without KS.

## Introduction

Immunosuppression and co-infections with certain oncogenic viruses appears to increase the risk of some cancers in HIV-infected patients [1]. Infection with Kaposi sarcoma herpes virus (KSHV) is a precondition for the development of Kaposi’s sarcoma (KS), and the seroprevalence of KSHV infection is high in both HIV-infected and uninfected populations, particularly in Africa [2]. Consequently, there has been a sharp increase in the incidence of KS since the advent of the HIV pandemic, and KS is a significant contributor to morbidity and mortality in sub-Saharan Africa [3]–[5]. Today KS is one of the most common cancers in Africa and is the most common tumour in HIV-infected individuals [4], [6]. In South Africa, the incidence of KS rose dramatically as the HIV epidemic escalated (increasing threefold between 1988 and 1996) [7]. KS has been found to be associated with advanced disease and high mortality among patients attending primary care clinics [8].

The World Health Organization (WHO) estimates that just over 5 million HIV-1-infected people were receiving antiretroviral therapy (ART) in sub-Saharan Africa by the end of 2010, for an estimated coverage of patients eligible for ART of 56% [9]. Combination ART has been used to successfully treat early stage KS for some time [10]–[12], achieving regression of KS lesions [13], [14] and successfully reducing KS-related mortality [13], [15]. In particular, suppression of replication of HIV has been associated with remission of KS [16]. In the United Kingdom, survival at 5 years among patients diagnosed with KS in the era of ART was estimated to be 98.4% in patients who had CD4 cell counts above 150 cells/mm^3^ and with skin or lymph node involvement only and no other symptomatic disease [17]. At the other end of the spectrum, 5-year survival was estimated at 8.4% in patients with a history of AIDS, more severe immunosuppression, more advanced KS and other symptomatic disease.

The prognosis of KS is not well defined in resource-limited settings and its influence on response to ART is unclear. In particular, the excess mortality related to KS among those on ART is not well described. We used cohort data from two large urban HIV care and treatment programs in Johannesburg and Cape Town, South Africa, to assess the effect of KS on survival, loss to follow-up and immunologic and virologic responses to ART.

## Methods

### Ethics Statement

This analysis was nested within ongoing cohort studies of routine ART outcomes at the sites in Cape Town and Johannesburg. Use of data from the Themba Lethu and Khayelitsha sites were approved by the Human Research Ethics Committee of the University of the Witwatersrand and the Ethics Committee of the University of Cape Town, respectively. The pooling of data in IeDEA-SA was approved by Ethics Committees at the Universities of Bern and Cape Town. Individual patient consent was not needed, consistent with the South African Medical Research Council’s Guidelines on.

Ethics for Medical Research and the Declaration of Helsinki. As this was a retrospective analysis of routine clinical service records, no additional data collection or procedures were undertaken from or on patients, all patient information was entered into the database using coded identification numbers, and no information that could reveal patient identity was available in the analytic datasets.

### Cohort Description

Data for this study came from two HIV treatment cohorts: the Themba Lethu Clinic in Johannesburg [18] and three clinics of the Khayelitsha ART programme in Cape Town [19], [20], South Africa. Both Themba Lethu and the Khayelitsha clinics are part of the International epidemiological Databases to Evaluate AIDS in Southern Africa (IeDEA-SA), a large collaboration of ART treatment programmes (www.iedea-sa.org) [21]. Themba Lethu has initiated over 16,000 patients on ART since its inception in 2004. The Khayelitsha clinics were set up by Médecins Sans Frontières (MSF) in 2001 and are now run by the Western Cape Provincial Department of Health. In August 2010, more than 16,000 patients were on ART. The data from the three Khayelitsha clinics were aggregated for this analysis. Care at all clinics was provided according to the South African National Department of Health guidelines in place during the study period [22]. Routine data were collected prospectively at each site to facilitate HIV treatment monitoring. Data from the different sites were transferred to the IeDEA-SA database managed by the Universities of Cape Town, South Africa and Bern, Switzerland.

### Eligibility Criteria

We included HIV-positive treatment naïve patients ≥18 years of age who initiated ART at a study clinic between 01 January 2001 and 31 December 2007. We limited the analysis to patients starting standard South African public sector first-line ART regimens (stavudine [d4T] or zidovudine [AZT] with lamivudine [3TC] and either efavirenz [EFV] or nevirapine [NVP]) [22]. During the study period, the National guidelines’ eligibility criteria for initiation of ART were either a CD4 cell count <200 cells/mm^3^ or a WHO stage 4 illness (such as KS) regardless of CD4 count. We found 13,847 patients were eligible for the current analysis.

### Study Variables

We compared ART outcomes by KS status at ART initiation. KS was defined as having a KS diagnosis recorded in the dataset between 6 months prior to and 6 months after ART initiation. KS is diagnosed mostly on a clinical basis at the study sites and while certain individuals may have had histopathological confirmation of disease, this is not routinely done in all cases. Our primary outcomes included: 1) all-cause mortality; 2) loss to follow up (LTFU); 3) failure to achieve virologic response at 6- and 12-months on ART (HIV viral load ≤400 copies/ml); and 4) failure to achieve immunologic response (CD4 count increase of >50 cells/mm^3^ at 6 months and >100 cells/mm^3^ at 12 months after ART initiation). LTFU was defined as having not attended the clinic in the previous 4 months. Mortality is ascertained through active tracing of patients who do not return to the clinic, and data for those lost was also verified at the end of 2010 with the South African National Vital Registration system for patients in whom a civil identification number was available (42% of those lost to care in Themba Lethu [23] and 47% in Khayelitsha [19]). As the hazard of mortality was not consistent over time, for both the mortality and LTFU outcomes, we considered the effect of KS on each of these events at any time point after initiation of treatment. We then further stratified the analysis into the first year after ART initiation and after the first year on ART.

### Statistical Analysis

Baseline characteristics for each group were stratified by KS status and summarized as proportions or medians with interquartile ranges. Cause-specific Cox proportional hazard models were used to estimate the effect of KS on mortality and loss to follow up on ART at each time period considered. Person-time was calculated from the date of ART initiation to the earliest of: 1) death or loss to follow up; 2) transfer to another facility; or 3) end of study period (31 December 2008). We used mixed linear models with a random intercept and an unstructured correlation matrix to estimate CD4 trajectories over time, accounting for repeated observations on an individual. Time was specified as a quadratic function. Models for those with KS and those without KS were fit separately to allow for different curves by exposure group. The association of KS with change in CD4 count from baseline to 6 and 12 months was estimated using a multivariate linear generalized estimating equation model. Additionally, log-binomial regression was used to estimate the impact of KS status on CD4 response (>50 cells/mm^3^ and >100 cells/mm^3^) and VL suppression (<400 vs. ≥400) by 6- and then by 12-months on treatment respectively. All models were adjusted for age, gender, baseline CD4 count (at ART initiation), tuberculosis treatment status, time period (year of ART initiation) and initiating treatment site.

## Results

A total of 13,847 patients met eligibility criteria, including 247 individuals (1.8%) diagnosed with KS at baseline (i.e. 6 months prior to up to 6 months after ART initiation). The prevalence of KS was slightly greater in Khayelitsha than at Themba Lethu (2.2% vs. 1.5%). The patient characteristics at initiation of ART are described in Table 1. Those with KS at ART initiation were similar to those without with respect to median age (35 vs. 35 years) and first-line ART regimen (68% vs. 69% initiated on d4T-3TC-EFV). The median presenting CD4 count was somewhat lower in KS patients (74 vs. 85 cells/mm^3^) but those with KS were about twice as likely to have a CD4 count in the 200–350 cells/mm^3^ category (12.3% vs. 7.2%). The proportion on TB treatment was also higher among those with KS (37% vs. 30%). As expected, patients with KS were more likely to be male than other patients (49% vs. 36%). Those without KS had received a median of 19.1 months of ART (IQR: 7.8–32.0) compared to 12.3 months (IQR: 2.3–29.8) among those with KS.

**Table 1:** Baseline characteristics of 13,847 adults initiating ART in Cape Town and Johannesburg, South Africa, stratified by presence of Kaposi sarcoma.

Characteristics		No Kaposi Sarcoma (n = 13,600)		Kaposi Sarcoma(n = 247)		
Sex	Male		4893 (36.0%)		121 (49.0%)	
Age at ART Initiation (years)	Median (IQR)		35 (30–41)		35 (30–41)	
Initiating treatment site	Khayelitsha		6583 (48.4%)		153 (61.9%)	
	Themba Lethu		7017 (51.6%)		94 (38.1%)	
Year of ART Initiation	Before 20042004		581 (4.3%)1947 (14.3%)		20 (8.1%)42 (17.0%)	
	2005		3185 (23.4%)		74 (30.0%)	
	2006		4149 (30.5%)		64 (25.9%)	
	2007		3738 (27.5%)		47 (19.0%)	
CD4 at ART Initiation (cells/mm^3^)	Median (IQR)		85 (33–150)		74 (29–152)	
	0–50		4256 (34.3%)		86 (37.9%)	
	51–100		2747 (22.1%)		46 (20.3%)	
	101–200		4518 (36.4%)		67 (29.5%)	
	200–350		899 (7.2%)		28 (12.3%)	
First-line ART Regimen	d4T/3TC/EFV		9200 (68.1%)		169 (69.3%)	
	d4T/3TC/NVP		3000 (22.2%)		52 (21.3%)	
	Other		1562 (11.7%)		23 (9.4%)	
TB at Initiation	Yes		3247 (29.5%)		71 (36.6%)	

TB = tuberculosis; IQR = interquartile range, ART = antiretroviral therapy; d4T = stavudine, 3TC = lamivudine, EFV = efavirenz, NVP = nevirapine.

Number of patients (%) are shown unless otherwise stated.

### Mortality and Loss to Follow Up

Vital status outcomes were ascertained for 13,065 (94%) of the 13,847 subjects (95% for those with KS and 94% for those without KS). Of these, 10% (1,312) died and 14% (1,837) were LTFU at some point after ART initiation (Table 2). Median follow-up time for those who died or were lost to follow up was 4.5 (IQR 1.5–12.5) and 9.6 (IQR 4.4–19.3) months, respectively. Mortality was highest within the first 12 months after starting ART (74% of deaths occurred in the first 12 months).

**Table 2:** The effect of Kaposi Sarcoma on mortality and loss to follow-up after initiation of ART among 13,065 adult HIV-infected patients initiating ART in Cape Town and Johannesburg, South Africa.

Death									Lost to follow up				
		Deaths	Person time (years)		Rate/100 pys*		CrudeHR (95% CI) †	AdjustedHR (95% CI)‡	LTFU**	Person time (years)	Rate/100 pys*	Crude HR (95% CI) †	Adjusted HR (95% CI)‡
***Over total follow-up***													
No KS	1248 (9.7%)		32345		3.9	1.0		1.0	1794 (14.0%)	32345	5.5	1.0	1.0
KS	64 (27.2%)		491		13.0	3.22 (2.51–4.15)		3.62 (2.71–4.84)	43 (18.3%)	491	8.8	1.58 (1.16–2.14)	1.42 (0.88–2.29)
***Within first year of ART***													
No KS	913 (7.1%)			12317	7.4	1.0		1.0	985 (7.7%)	12317	8.0	1.0	1.0
KS	54 (23.0%)			191	28.3	3.63 (2.76–4.77)		4.05 (2.95–5.55)	26 (11.1%)	191	13.6	1.69 (1.14–2.49)	1.55 (0.85–2.82)
***After first year of ART***													
No KS	335 (3.1%)			18745	1.8	1.0		1.0	809 (7.4%)	18745	4.3	1.0	1.0
KS	10 (6.5%)			244	4.1	2.03 (1.08–3.80)		2.30 (1.08–4.89)	17 (11.0%)	244	7.0	1.44 (0.89–2.32)	1.21 (0.54–2.70)

† HR = hazard ratio, CI = confidence interval, KS = Kaposi sarcoma, ART = antiretroviral therapy, pys = person years, hazard ratios from a Cox proportional hazards regression model.

‡ Models adjusted for sex, baseline CD4 count, age, treatment site, tuberculosis at ART initiation, year of ART initiation.

* pys = person years.

** LTFU = Lost to follow up defined as having missed a clinic appointment by at least 3-months after the scheduled visit date.

A greater proportion of individuals with KS died after ART initiation compared to those without KS (27% vs. 10%). The rate of LTFU after ART initiation was 13.0/100 py among those with KS compared to 3.9/100 py among those without KS. Individuals with KS had higher mortality rates at all durations after ART initiation compared to those without KS: 28.3/100 person-years (100 py) vs. 7.4/100 py within the first year and 4.1/100 py vs. 1.8/100 py after the first year.

**Figure 1:**
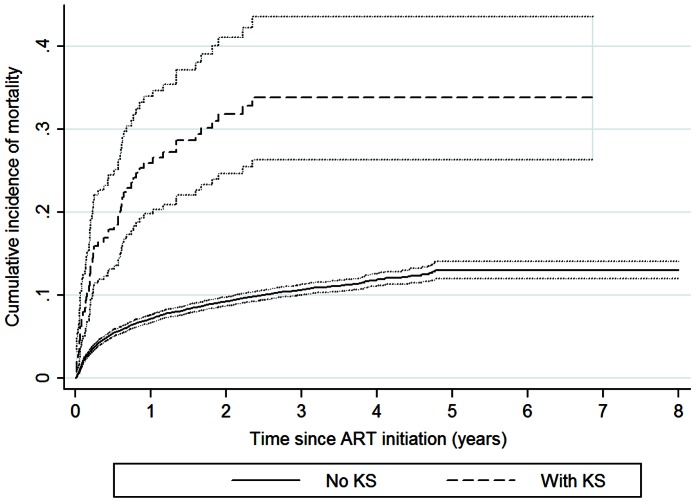
Cumulative incidence of mortality after ART initiation by KS status.

Cumulative incidence curves showed higher incidence of mortality for those with KS after ART initiation with the greatest differences in mortality occurring within the first year on treatment (Figure 1). The risk of death for those with KS was over three times that of those without KS at any time point after ART initiation (adjusted HR: 3.62; 95%CI: 2.71–4.84) and four times greater within the first year after ART initiation (adjusted HR: 4.05; 95%CI: 2.95–5.55) (Table 2). Among those who survived to a year on treatment, the risk of death was still greater in the KS group though the magnitude of this effect was smaller (adjusted HR: 2.30; 95%CI: 1.08–4.89).

We also analyzed the effect of time of KS diagnosis in relation to ART initiation on mortality. The mortality rate after ART initiation was greater among those diagnosed with KS before ART initiation than those diagnosed with KS after ART initiation (14.2/100 py vs. 9.8/100 py) though both of these were greater than the proportion who died among those without KS (3.9/100 py). The hazard of death among those diagnosed with KS before ART initiation was higher than the hazard among those diagnosed with KS after ART initiation (HR = 4.14 95% CI 2.97–5.77 vs. HR = 2.61 95% CI 1.47–4.62) comparing both groups to those without KS.

A greater proportion of individuals with KS were LTFU after ART initiation compared to those without KS (18% vs. 14%). The rate of LTFU after ART initiation was 8.8/100 py among those with KS compared to 5.5/100 py among those without KS. Among those with KS, the rate of LTFU was greatest in the first year after initiation of ART (13.6/100 py in the first 12 months vs. 7.0/100 py after 12 months). In adjusted proportional hazards models there was little evidence for a difference in the rate of LTFU in those with KS compared to those without, both in first year (adjusted HR: 1.55; 95%CI: 0.85–2.82) and after a year on treatment (adjusted HR: 1.21; 95%CI: 0.54–2.70). In competing risks analyses with death as the competing event, the rate of LTFU was closely similar but attenuated (HR: 1.02; 95% CI: 0.59–1.78).

### Immunologic and Virologic Failure

Among the 12,337 subjects alive and in care at 6 months on treatment, CD4 count values were available for 8,676 (70%) of these (63% of those with KS and 70% of those without KS). By 6 months on treatment, nearly a quarter of patients (23.7%; 95% CI: 17.3–32.7%) with KS had failed to achieve a CD4 increase of ≥50 cells/mm^3^ compared to 18.1% (95% CI: 17.5–19.1) of those without KS (Table 3). The median increase in CD4 count by 6 months on ART was 98 cells/mm^3^ (IQR 58–164 cells/mm^3^) among the KS group and 121 cells/mm^3^ (IQR 66–190 cells/mm^3^) for those without KS. Patients with KS gained, on average, 29 fewer CD4 cells (95% CI: 7–52 cells/mm^3^) than those without KS over the same time period. Among the 11,667 patients who survived to a year on treatment, CD4 count values were available for 7,157 (62%) of subjects. 29.9% (95% CI: 21.4–39.6%) of KS patients failed to achieve a 100 cell increase in CD4 count compared to 23.3% (95% CI: 22.3–24.3%) of patients without KS. The median increase in CD4 count by 12 months on ART was 150 cells/mm^3^ (IQR 90–225 cells/mm^3^) among the KS group and 175 cells/mm^3^ (IQR: 105–260cells/mm^3^) for those without KS.

**Table 3:** Immunologic and Virologic Outcomes at 6 and 12-months on ART stratified by KS status among 8,676 adult HIV-infected patients initiating ART in Cape Town and Johannesburg, South Africa.

6-months				12-months		
Exposure	Number with failure	Crude RR (95% CI)‡	Adjusted† RR (95% CI)‡	Number with failure	Crude RR (95% CI)‡	Adjusted† RR (95% CI)‡
***Immunologic failure*** *						
No KS	1565 (18.3%)	1.0	1.0	1655 (23.3%)	1.0	1.0
KS	29 (24.4%)	1.33 (0.97–1.83)	1.43 (0.99–2.06)	29 (29.9%)	1.28 (0.94–1.74)	1.20 (0.84–1.73)
***Failure to suppress HIV viral load*** **						
No KS	642 (7.8%)	1.0	1.0	714 (10.2%)	1.0	1.0
KS	14 (10.7%)	1.37 (0.83–2.26)	0.82 (0.38–1.79)	7 (6.9%)	0.67 (0.33–1.38)	0.25 (0.06–1.00)

† Models adjusted for sex, baseline CD4 count, age, treatment site, tuberculosis at ART initiation, year of ART initiation.

‡ VL = viral load, RR = relative risk, CI = confidence interval, relative risk from a log-binomial regression model KS = Kaposi’s sarcoma, ART = antiretroviral therapy,

* Failure to achieve a CD4 response defined as an increase of ≥50 cells/mm^3^ at 6 months and ≥100 cells/mm^3^ at 12 months.

** Failure to suppress VL to <400 copies/ml.

**Figure 2:**
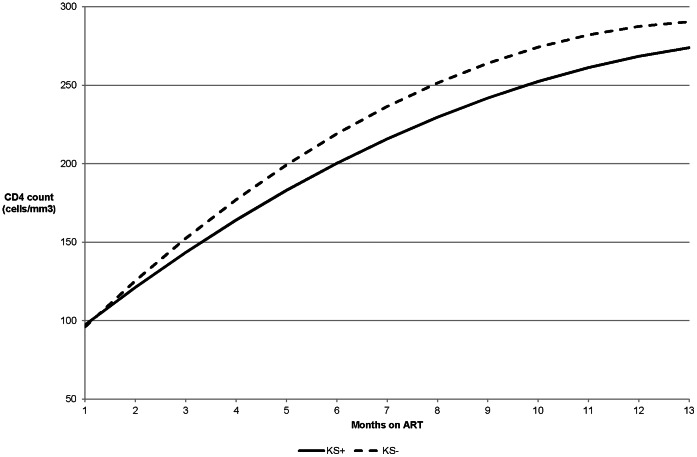
Mean predicted* CD4 cell count increase from ART initiation stratified by KS status. *Trajectories were estimated using two separate mixed linear models, one for the KS+ and one for the KS- to allow the curves to depart from being parallel. Curves were fitted using time as a quadratic function and a random intercept with an unstructured correlation matrix for repeated measures.

Using adjusted generalised estimating equations, those with KS gained an estimated 9 fewer CD4 cells (95% CI -21–40 cells/mm^3^) than those without KS over the first year of ART. The predicted CD4 trajectories from start of ART suggested some advantage for those without KS (Figure 2). Despite starting on very similar CD4 cell counts at ART initiation, those with KS gained fewer CD4 cells over the first year of treatment compared to those without KS. By the end of the first year the rate of increase in CD4 count was similar for the groups, though the group without KS retained consistently higher CD4 cell counts after treatment initiation. In log-binomial models, patients with KS were more likely to fail to achieve a 50 cells/mm^3^ increase (RR 1.43; 95% CI: 0.99–2.06) and 100 cells/mm^3^ increase (RR 1.20; 95% CI: 0.84–1.73) in CD4 count at 6- and 12-months on treatment respectively (Table 3). Sensitivity analyses yielded no qualitative differences in results when attributing either achieving or failing to achieve the outcome to all missing CD4 count responses at 6 or 12 months on treatment (results not shown).

Virologic response to ART was favourable among both groups (Table 3). By 6 months on treatment, only 11% of those with KS had failed to suppress HIV viral load to <400 copies/mL while just under 8% of those without KS had failed to achieve suppression. Among those who survived to a year on treatment, similar proportions failing to achieve virologic responses were noted (7% vs. 10%). The relative risk for failure to achieve virologic suppression suggests that KS patients fared better at 6 (RR: 0.82; 95% CI: 0.38–1.79) and 12 months (RR: 0.25; 95% CI: 0.06–1.00) after initiation of ART compared to those without KS though these estimates lacked precision. Results from sensitivity analyses again did not qualitatively change the results at either 6 or 12 months on treatment.

## Discussion

AIDS-related malignancies are increasing in significance as the HIV epidemic matures, particularly in resource-limited settings. These settings bear the greatest burden of disease due to cancers yet are most limited in terms of drug options, infrastructure and staffing required to effectively treat these conditions. As access to ART continues to scale-up and models of HIV care shift away from specialist services to decentralised clinics, investigating factors that impact on the effective response to ART is important. ART has shown much promise in the treatment of early stage KS [10]–[16], [24] but the effect of KS on response to ART is not clear.

We found the prevalence of Kaposi sarcoma among this HIV-infected treatment naïve population was 1.8%, very similar to the prevalence of 1.6% seen in Nigeria [25], and somewhat lower than seen among European cohorts (3.8–6.4%) in the late 1990s [26]–[28]. While there may be country-level differences in prevalence of KS, our figures could underestimate the true prevalence in this setting for several reasons. Firstly, diagnosis of KS in HIV outpatient clinics may be impaired by limited access to oncology and histopathology services, as well as lack of training on early recognition KS by primary care staff. A recent study in South Africa noted that 46% of study subjects were diagnosed with KS and HIV at the same time [29] and earlier studies have shown high pre-ART attrition underestimates KS prevalence; in South Africa the prevalence of KS including pre-ART subjects was estimated at 3.4% [8]. Additionally, limited communication and linkage between oncology and outpatient services hampers the recording of cancer diagnoses in HIV clinic patient records and use of national cancer registry databases to ascertain cancer diagnoses is not routine.

We demonstrated a substantially increased risk of mortality associated with KS. The risk of death was four times greater among the KS group in the first year on ART and though the risk decreased thereafter, those with KS were still twice as likely to die after the first year of treatment as those without KS after adjustment for measured confounders. This is in keeping with previous findings on KS mortality [8], [25] and highlights the importance of early diagnosis and initiation of appropriate treatment for HIV-infected subjects with KS at every stage of HIV infection and treatment. We also note that the KS group were more likely to have a diagnosis of tuberculosis at initiation of ART when compared to those without KS. Though TB is not diagnosed primarily by chest x-ray in these settings, the radiographic appearance of the nodular infiltrate associated with pulmonary KS could have been mistaken for TB in some cases. Pulmonary KS is associated with high rates of mortality and though all models were adjusted for diagnosis of TB at ART initiation, this may have contributed to the excess mortality noted in the KS group.

Though the results were imprecise and lacked statistical significance, we note that the majority of estimates suggested those with KS were less likely to fail to suppress HIV viral load. It is possible that this reflects survivor bias in that those with KS who are also poorly adherent to treatment do not survive to have a viral load test done at the intervals described. Though we cannot make inferences from our results, if this effect were real, it might suggest better adherence among those surviving with KS possibly related to more intensive follow up and more frequent attendance at clinic visits for their KS related care. We did also note some immunologic differences. First, those with KS were roughly twice as likely to have a nadir CD4 count between 200 and 350 cells/mm3 compared to those without KS. This is likely explained by the fact that KS (as a WHO stage 4-defining condition) was an indication for initiation of ART with CD4 count ≥200 cells/mm3 at a time when the ART eligibility criteria were otherwise <200. Second, after initiation of ART, those with KS were less likely to increase their CD4 cell counts by 50 and 100 cells at 6 and 12 months on treatment respectively. The KS group also had a smaller mean increase in CD4 cell count at both time periods than those without KS though the actual difference in CD4 gain was small between the groups. This may be due to differences in disease stage at treatment initiation [30] or possibly related to the additional suppressive effect of chemotherapy on the KS patients’ immune system. CD4 cell counts have been documented to decline by up to 50% during chemotherapy even in the presence of virally suppressive ART [31], an additional immune insult that would not be experienced by those not receiving chemotherapy and could partially explain the lack of significant difference in viral load suppression in this study. We emphasize that our findings apply only to those who remained alive and in care with follow-up laboratory results at the time points considered.

Our findings need to be considered in light of the study’s limitations. First, the prevalence of KS is likely to be underestimated in these cohorts due to the remote location of oncology services from the HIV outpatient clinics and other reasons explored above. Current linkage projects in collaboration with the National cancer registry are underway which aim to improve ascertainment of cancers among HIV treatment cohorts. Second, high rates of lost to follow up in these cohorts may introduce the possibility of selection bias, though we did not find significant differences in rates of LTFU between the groups. The rate of LTFU may, however, have led to underestimations of the mortality rates due to different mortality rates between patients lost to follow-up with and without KS. As previously noted, anywhere from 20–50% of those lost from HIV care are actually deceased [23], [32]–[34]. Finally, we lacked data on staging of KS disease and use of chemotherapy and were unable to adjust models for these or estimate the effect of chemotherapy and ART on treatment outcomes, particularly among those with visceral advanced KS.

Despite effective ART, clinical KS remains a poor prognostic factor in ART treatment outcomes. High rates of mortality and loss to follow up confirm that KS remains a significant problem in low and middle income countries. Insufficient awareness at primary care level leads to under-diagnosis of KS, especially in its early stages, and result in patients often presenting with advanced disease. Delays in initiation of treatment for AIDS-related malignancies such as KS remain a problem in settings where oncology services and chemotherapy are offered at specialist tertiary centres often remote from primary care services and unable to cope with the high patient burden. Under-diagnosis of disseminated disease and limited chemotherapy options are also likely to be negatively impacting on survival and need to be addressed to improve outcomes for HIV-infected patients presenting for treatment with KS. In particular, chemotherapeutic options, such as paclitaxel, are not readily available in resource-limited settings due to high cost. Future research efforts may focus on investigating alternate chemotherapy drugs, including etoposide [35]–[37], which could be safely administered at primary care level in order to increase access and reduce delays and resulting mortality.
